# A systematic review on the determination and analytical methods for furanic compounds in caramel models

**DOI:** 10.1007/s13197-025-06419-4

**Published:** 2025-08-19

**Authors:** Ai Chee Chan, Siti Umairah Mokhtar, Pui Khoon Hong

**Affiliations:** 1 Faculty of Industrial Sciences and Technology, Universiti Malaysia Pahang Al-Sultan Abdullah, Lebuh Persiaran Tun Khalil Yaakob, Gambang, Kuantan, 26300 Pahang Malaysia; 2 Politeknik Sultan Haji Ahmad Shah, Semambu, Kuantan, 25250 Pahang Malaysia; 3 Centre for Bioaromatic Research, Universiti Malaysia Pahang Al-Sultan Abdullah, Lebuh Persiaran Tun Khalil Yaakob, Gambang, Kuantan, 26300 Pahang Malaysia

**Keywords:** Caramel, Furanic compounds, 5-hydroxymethyl-2-furfural (HMF), Maillard reaction and analytical methods

## Abstract

Caramelization is the heat-induced transformation of sugars, leading to the formation of characteristic caramel flavours and colours. Furanic compounds such as 5-hydroxymethyl-2-furfural (HMF) and furfural were the intermediate products in this caramelization process. Excessive amounts of HMF, may pose food safety concerns, potentially influence human health. This systematic review aims to elucidate the types of sugar and amino acids that are usually used in previous studies, the type, and quantities of furanic compounds in the caramel model and to explore the diverse quantification methods employed in relevant studies. Preferred Reporting Items for Systematic Reviews and Meta-Analyses(PRISMA)method was employed in this study. Employing keywords such as “caramel”, “caramelization”, “browning”, “furanic”, “furfural”, “furan”, and “hydroxymethylfurfural” were used. A total of 1303 papers were obtained from the initial search by using Web of Science and Scopus database. Finally, 21 papers were selected after being evaluated. The results showed glucose and lysine were the most employed sugar and amino acids to develop caramel and Maillard reaction model in the previous studies. HMF emerged as the prevalent furanic compound, closely followed by furfural and 5-methylfurfural. These three furanic compounds contribute to the bitter taste of the caramel model. High Performance Liquid Chromatography (HPLC) is the preferred method for furanic compound analysis due to the low melting point and polar properties of furanic compounds. Other quantification methods include Solid-Phase Microextraction Gas Chromatography Mass Spectrometer (SPME GC-MS) and spectroscopy methods. This paper summarises furanic chemical analysis in caramel models, including existing understanding and methodological trends.

## Highlights

This is the first review article that focused on the furanic compounds in various types of caramels, the key furanic compounds highlighted include 5-hydroxymethylfurfural (HMF), furfural and 5-methylfurfural.

HMF is a prominent indicator of caramelization extend and thermal load, as well as assessing potential toxicity and monitoring of heat-induced contaminants in the caramels.

The furanic compound levels are influenced by pH, temperature, reaction time, along with the types and concentrations of carbohydrates and amino acids present in the reaction.

Detailed setup and results from the high-performance liquid chromatography (HPLC) and gas chromatography (GC) approaches for determining the quantity of furanic compounds are summarised in tables for ease of reference.

This review paper revealed significant discoveries, approaches, and areas of limited understanding regarding the analysis of furanic compounds in caramel models. As a result, it offers valuable perspectives to enhance caramelization procedures therefore elevating food quality, safety, and health.

## Introduction

Caramel is a substance produced at elevated temperatures from mono- and disaccharides carbohydrates. The heat turns the white crystalline sugar to brown colour, giving it a slightly bitter taste and aroma of burnt sugar which are both favourable improvements in food. Caramel is commonly used for food flavourings and colourings, such as frozen desserts, gravies, sauces, marinated meats, spirits, wines, beers, flavoured sodas, and chocolate (Kuhnert 2015; Xu et al. 2023). These compounds may have other functional effects besides used as food colours and flavouring. For example, caramel could emulsify and stabilise colloidal systems, helping to preserve flavour and disperse water-insoluble substances such as essential oils. In addition, the caramel in some beverages is to delay flavour changes caused by sun exposure (Vollmuth 2018).

Depending on the materials used in its manufacturing process, caramel is categorized into four classes: Class I is plain caramel, Class II is sulphite caramel, Class III is ammonia caramel, and Class IV is sulphite ammonia caramel. Wine and beer are frequently made with class I and class II caramels. Class III caramel is frequently found in candy, beer, and sauces. Soft drinks often contain class IV caramel (Li et al. 2022). There are three different interpretations for caramel: (i) solid or semi-solid used to make sweets and decorate cakes and pastries; (ii) syrup used to flavour sauces and various beverages, and (iii) liquid used in certain foods and medicine (Vollmuth 2018).

Caramelization and the Maillard reaction are the two types of non-enzymatic browning that can happen in foods. Both reactions involve the application of heat to food, resulting in browning and the development of complex flavours. They are essential processes in cooking and food preparation, contributing to the sensory attributes of a wide range of dishes (Abrantes et al. 2022; Xu et al. 2023).

Caramelization involves isomerization, epimerization, dehydration, and oxidation through enolization. 1,2-enediol, a frequent intermediate, isomerizes aldose and ketose sugars. The isomerization changes the structure of C-2 in aldoses and C-3 in ketoses by epimerization. Dehydration of sugars produces several reactive deoxyosones, which are crucial for the development of flavour and colour in caramel. Dehydration of deoxyosones will produce furanic compounds such as furans, furfural, 5-methylfurfural and 5-hydroxymethyl-2-furfural (HMF) (Mori and Ito 2004).

In Maillard reaction, sugar interacts reversibly with the amino compound to create a Schiff base. The Schiff base rearranges to produce Amadori and Heyn’s products. These products then degraded to furanic compounds in acid condition and dicarbonyl compounds in alkaline condition. High molecular weight products, such as melanoidin, are created by further interaction of sugar and amino groups. The various hues attributed to caramel colour are mainly produced by melanoidin (BeMiller 2019).

**Fig. 1:**
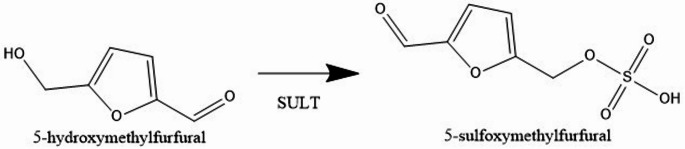
Conversion of HMF to SMF in living organisms

Furanic compounds are heterocyclic organic compounds containing a furan ring. HMF are frequently used to determine the thermal load of processed foods. Due to its carbonyl group, HMF may also be recognized as a reactive intermediate. Conversion of HMF to 5-sulfoxymethylfurfural (SMF) is the primary concern in food safety. Scientists have found that sulfotransferases (SULT) can change HMF into SMF by sulfonating its allylic hydroxyl functional group. Figure 1 shows the conversion of HMF to SMF. Sodium sulphate is a good leaving group in SMF which creates a very reactive intermediate that can combine with DNA or other molecules, leading to harmful and genetic effects (Capuano and Fogliano 2011). SMF is not stable, but it was found in the blood of mice that had been treated with HMF. This shows that HMF is metabolized in living organisms into SMF (Monien et al. 2009).

The European Food Safety Agency concluded that the mutagenicity of SMF provides sufficient evidence to raise toxicity and mutagenicity, (EFSA 2005). However, HMF is safe when used as directed, this result also applies to 5-methylfurfural, since 5-methylfurfural can be broken down into HMF (EFSA 2011). Consumption over the recommended level of HMF can cause mutagenic, carcinogenic, organotoxic and enzyme-inhibitory effects (Shapla et al. 2018; Vollmuth 2018).

European Food Safety commission suggested that consumption of HMF should not be more than 1.6 mg/day (Liu et al. 2021). Besides, the European Commission has established an HMF threshold concentration of 40 mg/kg for general type of honey (European Commission 2001).

Others furanic compounds such as 2-furfural and 5-methylfurfural, recognized as bitter compounds. Bitter compounds in excess are denied due to their association with toxicity and the negative impacts that they have on digestion (Li et al. 2019). For 2-furfural, Adams et al. (1997), suggested that the maximum level of 2-furfural in ready to eat food like gravies are at 4.2 mg/kg and meat products are at 63.2 mg/kg.

Both Maillard reaction and caramelization enhance brown colour, flavour, aroma, and antioxidant properties. In contrast, these reactions can cause nutrient loss of proteins and the formation of complex compounds such as pyrazine, α-dicarbonyl and furanic compounds. Furanic compounds are intermediates associated with desired volatile or aromatic compounds, cellular and tissue dysfunction, and disease (Ozgolet et al. 2022). Thus, the study of non-enzymatic browning reaction in the caramel model where sugars and amino acids react under a stated condition can predict the browning process in its early and final stage of food processing (Carabasa-Giribet and Ibarz-Ribas 2000). While previous research has analyzed furanic compounds in caramel models, there has been no systematic evaluation synthesizing the types of models used, the range of furanic compounds detected, and analytical methods employed across studies; this review aims to comprehensively consolidate and critique these aspects to inform future work exploring furanic compound analysis in caramelization.

The significance of studying furanic compounds in caramel models extends beyond flavour development. As caramelized products are widely used in the food industry, understanding the formation and presence of furanic compounds is crucial for ensuring food safety and quality.

## Methodology

### Research questions

This systematic literature review (SLR) reviews the issue based on several research questions as follows: What kind of sugars and amino acids are studied for caramel? What type and how many furanic compounds are found in caramel models? What is the most common method to determine the furanic compounds in caramel models?

### Research inclusions and exclusions

In this study, the time range for the research article was published, starting from 2000 until October 27, 2023. This study’s inclusion criteria included research on the detection method and composition of furanic compounds in caramels made from various types of carbohydrates and amino acids. This investigation included commercial caramel with stated classes of caramel, and carbohydrates, which react with amino acids only. The carbohydrate reacts with peptides or proteins, and caramelization of food was excluded. Manuscripts that did not report the amount of furanic compounds were excluded. Furthermore, review studies, book chapters, and articles in languages other than English were excluded.

### Searching procedure

**Fig. 2:**
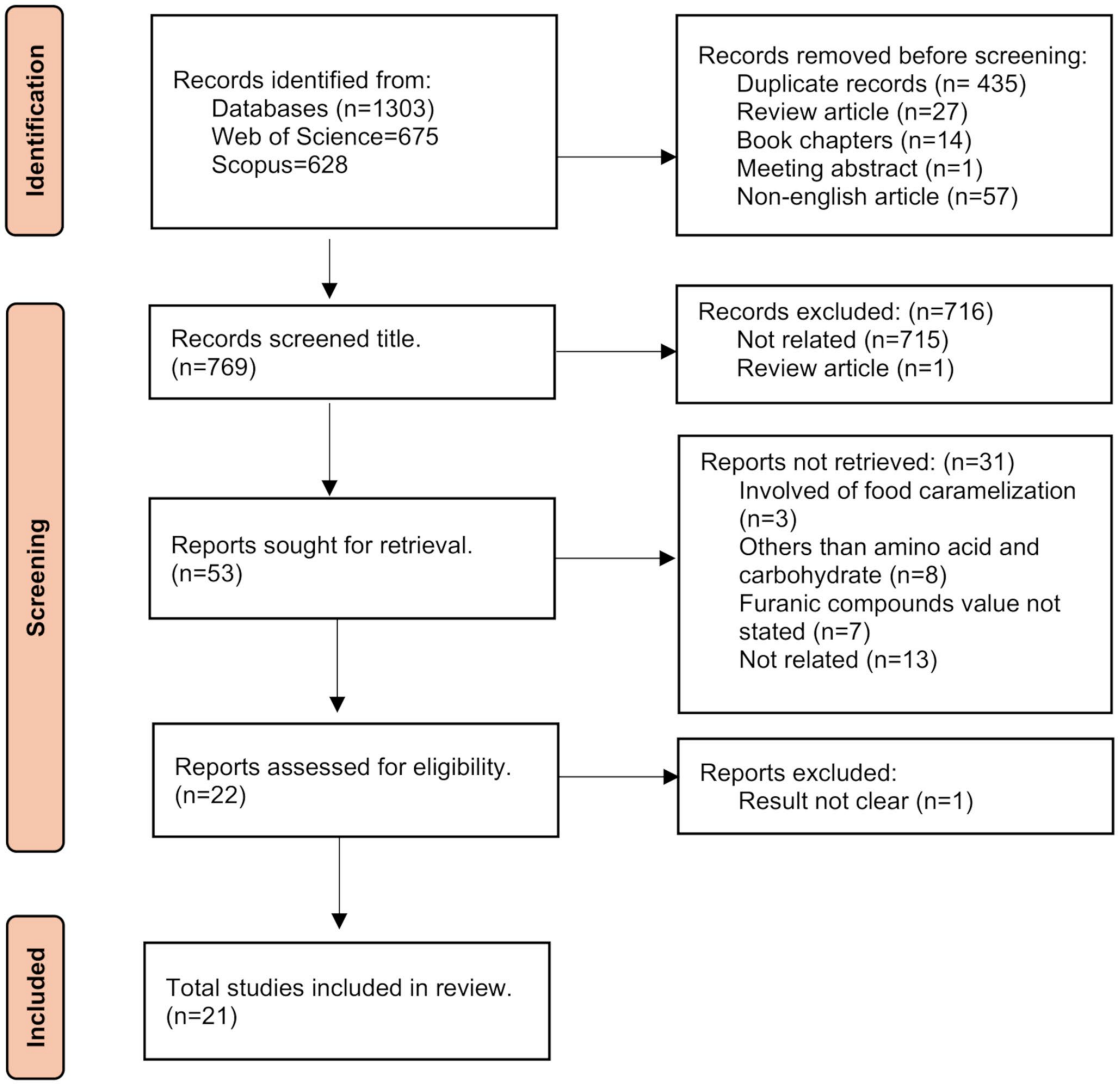
Flow diagram of search process

First, it was examined in Web of Science and Scopus databases to ensure no review study was published in this area. Keywords such as (caramel OR caramelization OR browning) and (furanic OR furfural OR furan OR hydroxymethylfurfural) were chosen. In the Web of Science and SCOPUS databases, keywords were searched in the title, abstract and keywords only. The search was completed on October 27, 2023. The complete searching procedure can be seen in Fig. 2.

### Steps of identification and selection publications

The first step was to get all the related research journals and conference papers. Following that, the duplicates were removed. The remaining studies were checked against the guidelines for inclusions and exclusions by referring to their titles and abstracts.

### Extraction of data

The criteria such as the composition of caramel models, amount of furanic compounds in different caramel models and the methodologies employed in evaluating furanic compounds were recorded and compiled in Tables 1, 2 and 3.

**Table 1:** The analytical method used for quantification and range of furanic compounds in various type of caramel

Caramel model	Reaction Conditions	Analytical method	Range of furanics compounds	Reference
Glucose + aspartic acid / glutamic acid / asparagine	85–100 °C, 80 h	UV/Visible spectrophotometer	HMF: 50- 1100 mg/L	Carabasa-Giribet and Ibarz-Ribas (2000)
Model 1 Maltose with L-proline Model 2 Maltulose / maltose with or without L-proline	Model 1 pH 8.0, 90 °C, 24 h Model 2 constant pH 4.0–8.0, 90 °C, 2 h	GC-MS	Model 1 2-furanmethanol: 0–90 $\:\mu\:$ mol/mol HMF: 0–50 $\:\mu\:$ mol/mol Model 2: 2-furanmethanol: 280- 650 $\:\:\mu\:$ mol/mol 2-furfural: 10- 170 $\:\:\mu\:$ mol/mol	Mori and Ito (2004)
Xylose / arabinose / fructose / glucose / sucrose + lysine	Model 1 irradiated (0–30 kGy) Model 2 80 °C, 4 h	HPLC–Diode array detector	Model 1 HMF and 2-furaldehyde: nd Model 2 HMF and 2-furaldehyde: 0.12–0.29 mg/L	Oh et al. (2005)
Fructose	pH 3.0, 75–95 °C, 80–120 h	HPLC-UV/Visible detector	HMF: 3.13- 90 mg/g	Chen et al. (2009)
Glucose + glutamic acid + antibrowning agent	pH 6.5, 50 °C, 48 h	HPLC-UV/Visible detector	HMF: 3.73–6.27 mg/L	Lim et al. (2010)
Glucose + ammonium sulphite	140 °C, 60 min	HPLC-Diode Array Detector	HMF: 15–65 mg/L	Guan, Zhu et al. (2011)
Glucose + ammonium sulphite	pH 7.5, 150 °C, 60 min, 0–18 Mpa	HPLC-Diode Array Detector	HMF: 3–76 mg/L	Guan, Yu et al. (2011)
Fructose with or without dietary polyphenols	pH = 7.0 or 10.0, 120 °C, 2 h	HPLC-Diode array detector	HMF: 100–1200 mg/L Furfural: 17–38 mg/L	Zhang et al. (2013)
Sucrose + citric acid + fiber	130 °C, 1 h	HPLC-Diode array and multiple wavelength detector.	HMF: 0.007–0.097 mg/L	Dogan and Toker (2015)
Sucrose + glycine	pH 1.0–4.0, 160–240 °C, 50s	HPLC–Diode array detector	HMF: < 0.02–1.11%w/w	Huang et al. (2016)
Glucose with or without Nacl	160–200 °C, 30 min	UFLC- Diode array detector	HMF: 0.10- 2.00 µmol	Kocadaǧll and Gökmen (2016)
Maltose / D-glucose + L-alanine / L-proline / L-lysine	pH 5, 130 °C, 300 min	HPLC-Diode array detector	HMF: 487–6518 µM Furfural: 27–143 µM	Kanzler et al. (2017)
Glucosamine	pH 7.0, 50–70 °C, 12 h	HPLC–Diode array detector	HMF: 2.1–6.2 mg/L	Dhungel et al. (2018)
Fructose + histidine	pH 3.0–9.0, 50–90 °C, 5 days	HPLC-Diode array detector	HMF: 0.089- 329.938 mg/L	Liu et al. (2019)
Commercial caramel	Class I, class III and class IV	QuEChERS Coupled -HPLC-DAD-MS	HMF: 11.0- 1058.1 mg/kg Furfural: nd- 10.2 mg/kg 5-methylfurfural: nd- 28.2 ± 1.5 mg/kg	Li et al. (2019)
Glucosamine + arginine/ lysine/ glycine/ cysteine/ proline/ serine/ methionine/ histidine/ threonine/ alanine/ valine/ leucine	pH 7.40, 70 °C, 12 h	HPLC–Diode array detector -MS	HMF: 6.4–58.7 mg/L	Dhungel et al. (2020)
Glucose + arginine	pH 9.0, 53–100 °C, 10–350 min	HPLC-Diode array detector	HMF: nd- 2.82 mg/L	Sogut et al. (2021)
Xylose + lysine	pH 6.5 or 8.0, 100 °C, 0.5–6 h	HPLC-Diode array detector	HMFO: 0.6 mM- 1.9 mM	Nakamura et al. (2021)
Glucose / fructose / sucrose + glycine / lysine / arginine with or without gallic acid	pH 5.0, 125 °C, 6 h	HPLC-Diode array detector	HMF Suc-Arg + GA (360 min): 19 mg/L Glu-Gly + GA (300 min): 178 mg/L	Abrantes et al. (2022)
Maltose / sucrose	140–180 °C	GC-MS	HMF: 86.78- 673.45 mg/L Furfural: 0.93–41.33 mg/L	Li et al. (2022);
Sucrose + NaCl / KCl	185 °C	SPME GC-MS	HMF: 1.2–2.2 mg/kg Furfural: 4.2–5.1 mg/kg 5-methylfurfural: 0.33–0.52 mg/kg	Xu et al. (2023)

**Table 2:** Summary of HPLC method for determination of furanic compounds in caramel model

Preparation method	Column	Detector	Detection wavelength (nm)	Mobile Phase	Gradient program	Flowrate (mL/min)	Detection limits	Reference
Direct inject.	C18	Diode array detector	284	A: acetonitrile B: water	Isocratic: A: B = 5:95	1.0	-	Oh et al. (2005)
Dilute with water.	C18	UV-visible detector	280	A: acetonitrile B: water C: acetic acid	Isocratic: A: B:C = 10:89.5:0.5	1.0	-	Chen et al. (2009)
Dilute with water.	C18	UV-visible detector	285	A: water B: acetonitrile	Isocratic: A: B = 9:1	1.0	-	Lim et al. (2010)
Dilute with water 1000 times.	dC18	Diode array detector	284	A: water B: methanol	Gradient elution	0.5	-	Guan, Zhu et al. (2011); Guan, Yu et al. (2011); Huang et al. (2016)
Direct inject.	C18	Diode array detector	280	A: acetonitrile B: 0.1% formic acid	Isocratic: A: B = 5:95	1.0	-	Zhang et al. (2013)
Dilute with water 400 times and centrifuge at 9000 rpm for 10 min.	C18	Diode array detector	284	A: 1% acetic acid B: acetonitrile	Isocratic: A: B = 95:5	1.0	-	(Dogan and Toker 2015)
Dilute with water.	dC18	Diode array detector	285	A: 10 mM formic acid B: acetonitrile	Isocratic: A: B = 90:10	1.0	-	Kocadaǧll and Gökmen (2016)
Dilute with water.	C18	Diode array detector	285	A: tetrabutylammonium hydrogensulfate and disodium edetatein phosphate buffer B: methanol	Gradient elution	0.5	-	Kanzler et al. (2017)
Direct inject.	C18	Diode array detector & MS	285	A: 0.05 M potassium dihydrogen phosphate and 0.005 M sodium octane sulfonate B: methanol.	Isocratic: A: B = 92.5:7.5	0.5	LOD HMF: 0.7 µg/mL LOQ HMF: 2.1 µg/mL	Dhungel et al. (2018); Dhungel et al. (2020)
Direct inject.	C18	Diode array detector	285	A: water B: methanol	Isocratic: A: B = 90:10	1.0	-	Liu et al. (2019)
Dilute with water 50 times and extract with QuEChERS method. Cleanup with C18 cartridge (SPE).	C18	Diode array detector & MS	284	A: water B: methanol	Gradient elution	1.0	Recovery HMF:101.5% Furfural: 89.4% 5-MF: 88.5% LOD HMF:15.0 µg/kg Furfural: 35.0 µg/kg 5-MF: 8.0 µg/kg LOQ HMF:50.0 µg/kg Furfural: 100.0 µg/kg 5-MF: 20 µg/kg	Li et al. (2019)
Dilute with water.	C18	Diode array detector		A: methanol B: 0.5% aqueous formic acid.	Isocratic A: B = 10:90	0.8	-	Sogut et al. (2021)
Direct inject.	C18	Diode array detector	320	A: 0.1% aqueous trifluoroacetic acid: methanol (98:2) B:0.1% aqueous trifluoroacetic acid: methanol (50:50)	Gradient elution	1.0	-	Nakamura et al. (2021)
Direct inject.	C18	Diode array detector	280	A: 0.3% formic acid aqueous solution B: methanol	Isocratic A: B = 90:10	1.0	-	Abrantes et al. (2022)

**Table 3:** Summary of gas chromatography method for determination of furanic compounds in caramel model

Preparation method	Column	Detector	Parameter details	Run time (mins)	Detection limits	Reference
Dilute with water 10 times. pH of adjust to 2.0 or 4.5. Treat with SPE techniques.	HP-FFAP wax column	MS in SIM mode	Splitless mode: Carrier gas: Helium Flow rate: 42 cm/s Inlet temperature: 200 °C Split mode: Ratio: 10:1 Carrier gas: Helium Flow rate: 40 cm/s Inlet temperature: 230 °C.	Splitless mode: 53 Split mode: 49	Recovery 2-Furfural: 88.8% 2-Acetylfuran: 90.3 Furanmethanol: 89.5% 2-hydroxyacetylfuran: 96.8% Furoic acid: 76.2% HMF: 101% Furaneol: 100.3%	Mori and Ito (2004)
Dissolved in 10% alcohol (1.0 g/mL)	HP-5 MS column	MS	Carry gas: Helium. Flow rate: 1.0 mL/min	34.35	-	Li et al. (2022)
Extraction with SPME (50/30 µm DVB / Carboxen / PDMS).	ZB-WAX column	MS	Inlet temperature: 200 °C. Spitless mode Carrier pressure = 103 kPa	46.5	-	Xu et al. (2023)

## Discussion

### Caramel models in previous studies

According to Table 1, most of the caramels were Maillard based caramel, where the sugar was reacted with amino acids. From the result, the most common carbohydrates studied for caramel models are glucose, followed by sucrose and fructose. Lysine is the predominant amino acid, followed by glycine, proline, and arginine. Lysine was likely selected due to its high reactivity related to its functional groups (Ajandouz et al. 2001). It is also an essential amino acid in human diets, demonstrating a food protein’s biological value (Oh et al. 2005).

Glucose paired with aspartic acid, glutamic acid and asparagine was chosen to model apple juice compounds (Carabasa-Giribet and Ibarz-Ribas 2000). Meanwhile, glucose and glutamic acid were utilized in models simulating soybean paste (Lim et al. 2010). Fructose was selected to represent its presence in fruits (Chen et al. 2009).

Caramel models were prepared at temperatures ranging from 50 to 240 °C. Lower temperatures 50–90 °C were more commonly applied, followed by 180 °C, and reaction times spanned 50 s to 5 days. High-temperature models were heated for shorter durations, while low-temperature models required longer heating times. Some studies adjusted the initial pH from 1.0 to 10.0; pH 7.0 represented a neutral and preferred model. The pH was held constant during heating only in the research of Mori and Ito (2004).

### Quantities of furanic compounds

Caramelization generates intermediates, including α-dicarbonyl and furanic compounds. Among furanic compounds, HMF was the predominant analyte across studies, with furfural second. Key motivations for quantifying furanic were developing detection methods (Li et al. 2019), analysing their flavour and aroma contributions (Li et al. 2019, 2022; Xu et al. 2023); utilizing HMF as an indicator of caramelization extent (Carabasa-Giribet and Ibarz-Ribas 2000; Chen et al. 2009; Kocadaǧll and Gökmen 2016) and thermal load (Cämmerer et al. 1999; Chen et al. 2009); assessing potential toxicity (Guan et al. 2011a; Dogan and Toker 2015; Huang et al. 2016); and monitoring heat-induced contaminants (Zhang et al. 2013; Dhungel et al. 2018).

In a glucose caramel model (30%), aspartic acid, glutamic acid and asparagine were added. Glucose-aspartic acid exhibited the highest HMF at 1100 mg/L, emphasizing the impact of temperature and duration. Glucose-glutamic acid showed the least HMF at 390 mg/L, revealing glutamic acid’s lower reactivity (Carabasa-Giribet and Ibarz-Ribas 2000). In the glucose-arginine model (pH 9), lower temperature (53 °C, 180 min) resulted in the highest HMF concentration (2.82 ± 0.12 mg/L), contrasting with the absence of HMF at higher temperatures (92–100 °C) (Sogut et al. 2021). HMF can undergo reduction and fragmentation reactions at high temperatures (100–160 °C) to produce 5-methylfurfural and furfural, respectively (Xu et al. 2023).

Another glucose model (1.25 M, 125 °C, pH 5.0), glucose-glycine (360 min) displayed the highest HMF at 152 mg/L compared to glucose-arginine (360 min) at 100 mg/L and glucose-lysine (60 min) at 140 mg/L. However, HMF accumulation decreased over time in the glucose-lysine model to 20 mg/L at 360 min. In the same study, the glucose model showed the highest HMF, followed by fructose and last sucrose in the first 60 min. Gallic acid effectively reduced HMF in the glucose-arginine and sucrose-arginine models, with notable HMF increases in glucose-lysine, glucose-glycine, and fructose-arginine models (Abrantes et al. 2022).

The glucose-glutamic model, treated with antibrowning agents, exhibited higher HMF in the storage at 30 °C than at 4 °C. In this study, antibrowning agents like cysteine and glutathione gave the lowest HMF in the glucose-glutamic model in most storage conditions but not at 30 °C in air. Citric acid and oxalic acid consistently inhibited HMF across storage conditions. (Lim et al. 2010). Higher ammonium sulphite and pressure significantly decreased HMF in glucose class IV caramel. The higher the ammonium sulphite added, the lower the HMF in caramel (decrease from 65 − 15 mg/L) (Guan, Zhu et al., 2011). When pressure is at 0 Mpa, HMF showed 76 mg/L and decreased to 33 mg/L at 18 MPa (Guan, Yu et al., 2011).

In a study involving glucose or maltose with alanine at pH 5 and 130 °C, glucose-alanine showed lower HMF (1210 µM) than maltose-alanine (2064 µM) (Kanzler et al. 2017). Meanwhile, a glucose-lysine model (80 °C, 4 h) exhibited the lowest furanic compounds (HMF and 2-furaldehdye) if compared to fructose-lysine and xylose-lysine models. Xylose-lysine showed the highest furanic compound at 0.29 mg/L (Oh et al. 2005). Other research for the xylose-lysine model found that the enormous amount of 4-hydroxy-5-methyl-3(2 H)-furanone (HMFO) was at 1.9 mM (pH 6.5) and 0.6 mM (pH 8.0), revealed pH-dependent variations in HMFO which stable in the model of pH 6.5 (Nakamura et al. 2021).

Sucrose models, exposed to varying pH and temperature, demonstrated increased HMF with higher temperatures and lower pH. As the temperature increased from 160 to 240 °C, and pH decreased from 4.0 to 1.0, HMF increased from 0.49% to 1.11% (Huang et al. 2016). Sodium chloride (NaCl) and potassium chloride (KCl) addition in a sucrose model increased furanic compounds. NaCl created more furanic compounds than KCl. At the same concentration of salts, HMF in the sucrose-NaCl model was 2.2 mg/L, and in the sucrose-KCl model was 1.2 mg/L (Xu et al. 2023).

In 2022, furanic compounds were studied as flavour compounds in the sucrose and maltose class I model. Total furanic compounds, especially HMF, were higher in sucrose than maltose model. Among 16 furanic compounds, the highest HMF at 673.45 mg/L was found in sucrose caramel and 86.78 mg/L in maltose caramel (Li et al. 2022). Besides of HMF, Li et al. (2022) also studied others furanics compounds, such as methyl-2-furoate, furfural, 5-methyl-2-furancarboxaldehyde and others. Li et al. suggested that sucrose caramels may impart a greater variety of flavours than maltose caramels.

Chen et al. (2009) studied fructose-based caramel (pH 3.0, at temperature of 75–95 °C) and found that HMF increased when the concentration of fructose increased. In the 1.11 M fructose system, HMF increased from 3.13 mg/mL (75 °C) to 56.10 mg/mL (95 °C). Zhang et al. (2013) studied the fructose model (pH 7 and 10) with dietary polyphenols. Chlorogenic acid dramatically increased the HMF value; however, rosmarinic acid effectively reduced the HMF in the fructose model. The fructose model (pH7) showed 550 mg/L HMF, fructose with chlorogenic acid showed 1200 mg/L and fructose with rosmarinic acid showed 350 mg/L, respectively. In the study of Liu et al. (2019), HMF increased when temperature, reactant concentration and fructose content increased; HMF decreased when histidine content and initial pH value increased. Based on Liu et al., the highest HMF was at pH 3.0 (329.94 mg/L), and the lowest HMF was at 0.16 M histidine (0.089 mg/L).

For the maltose-proline model (pH 8.0), pH dropped to 4.5 after 24 h, producing 2-furanmethanol at 90 µmol/mol and HMF at 50 µmol/mol. Then, they prepared maltose with or without proline, and pH was constant when incubating. Under pH 4.0, they found almost no volatile compounds except HMF formed in small amounts. 2-furan-methanol began forming at pH 5, and the most significant amount was at pH 8.0, 440 µmol/mol for 2-furan-methanol (Mori and Ito 2004). In another study, maltose reacted with proline, alanine, or lysine at 130 °C (pH 5) for 300 min. Maltose-proline showed the lowest HMF at 487 µM, maltose-lysine showed the highest HMF at 6518 µM, and maltose alone showed HMF at 1694 µM. The highest furfural formed in maltose-alanine at 143 µM and the lowest in maltose-proline at 27 µM. In this study, proline reduces the HMF and furfural compounds in the maltose caramel model (Kanzler et al. 2017).

Glucosamine models, studied under different conditions, highlighted the impact of vacuum and amino acids on HMF levels. A sous-vide glucosamine model (15%, 50–70 °C,12 h, pH 7.0) showed the highest HMF in non-vacuum treatment at 70 °C (6.2 mg/L) and lowest HMF at vacuum condition at 50 °C (2.1 mg/L). Vacuum conditions and lower temperatures could reduce the formation of HMF (Dhungel et al. 2018). Glucosamine (15%, 70 °C, 12 h) studied with twelve types of amino acids showed the highest HMF in glucosamine-arginine, around 58.7 mg/L. No amino acids could reduce the HMF amount in the glucosamine model in this study (Dhungel et al. 2020).

Commercial caramel analysis revealed Class IV caramel containing the high HMF, furfural, and 5-methyl furfural. Class I caramel exhibited low levels for HMF, furfural, and 5-methyl furfural. Class IV caramel contained 1058.1 mg/kg HMF, 10.2 mg/kg furfural and 28.2 mg/kg 5-methylfurfural while class I caramel contained 11.0 mg/kg HMF and around 0.5 mg/kg furfural (Li et al. 2019).

The wide variation in furanic compounds formed depends on the concentrations and types of reactants, pH, temperature, reaction time and other conditions. Varying preparation procedures limit the comparison of furanic compounds levels among different caramel models.

### Analytical methods

There are a few methods used to analyse furanic compounds in caramel. According to Table 1, the methods included a spectrophotometer, liquid chromatography, and gas chromatography. High-Performance Liquid Chromatography (HPLC) predominated for quantifying compounds in solution, while Gas Chromatography (GC) enabled analysis of volatile furanics (Mori and Ito 2004; Li et al. 2022), including recent Solid-Phase Microextraction (SPME) approaches (Xu et al. 2023). UV-Vis spectrophotometry was rarely applied in the caramel model (Carabasa-Giribet and Ibarz-Ribas 2000).

#### High-performance liquid chromatographic (HPLC) analysis

Furanic compounds such as HMF were mainly determined by the HPLC method. It is a suitable method to analyse water-soluble furanic compounds such as HMF, which is a key compound in caramel samples. HMF is soluble in water; the boiling point is 115 °C, and the melting point is 31.5 °C (Martins et al. 2022). The summary of the HPLC methods used by previous studies is tabulated in Table 2.

For HPLC system, the caramel sample was either diluted with water or directly injected after filter with 0.45 μm–0.22 μm pore size filter; with no derivation needed. For other sample preparation methods, Dogan and Toker (2015) centrifuged their sample at 9000 rpm for 10 min and Li et al. (2019) extracted the sample using the Quick, Easy, Cheap, Effective, Rugged and Safe (QuEChERS) method and treated it with Solid Phase Extraction (SPE) in a C18 cartridge before injecting it in HPLC system. QuEChERS is a straightforward extraction method that consists of an initial partitioning followed by SPE cleanup. QuEChERS is user-friendly and efficient, requiring only laboratory glassware and small quantities of organic solvents and samples. Furthermore, it adheres to the principles of green chemistry (Perestrelo et al. 2019). SPE is a suitable method to purify food that contained the complex matrix. QuEChERS demonstrated an excellent recovery for HMF, where 107.3% HMF was recovered, followed by 5-methylfurfural (91%) and furfural (86%).

According to Table 2, silica-based and reverse-phase C18 columns are commonly employed to analyse furanic compounds in caramel. These types of columns can separate and analyse furanic compounds efficiently. The retention time range for HMF in HPLC was from 6.5 to 31 min and UV-visible detector was prevalent over MS detection. UV-visible detector is able to detect furanic compounds based on their characteristic absorption wavelength which commonly was 280–285 nm, and HMFO at 320 nm. The maximum absorption wavelength of HMF is 284 nm (Hudz et al. 2019).

Polar mobile phases, such as acetonitrile, methanol and water, or acidified water, were commonly applied in HPLC system for furanic compounds analysis. This polar mobile phase dissolved well for the HMF and furfural compounds. The flow rate of the mobile phase was set to 0.5- 1.0 mL/min, and the total run time ranged from 15 to 70 min. Dhungel et al. (2018) optimized the mobile phase consisting of potassium dihydrogen phosphate, sodium octane sulfonate and methanol, achieving a limit of detection (LOD) at 0.7 µg/mL and a limit of quantitation (LOQ) at 2.1 µg/mL.

For the HPLC analysis, sample preparation steps such as filtration, or extraction will add complexity and potential sources of error or losses. For the complex caramel sample matrix, which contaminants might interfere in the analysis and thus may require additional sample clean-up step which is SPE (Gan et al. 2023). It requires relatively long-time analysis of up to 70 min, which may limit the throughput for large number of samples.

#### Gas chromatography method

The summary of the GC method applied by previous studies is shown in Table 3. Almost all GC method used Mass Spectrometer to identify and detect furanic compounds which are the important flavour or volatile aroma compounds in the caramel model (Mori and Ito 2004; Xu et al. 2023). GC-MS enabled selective, sensitive quantitation of volatile furanic compounds (Martins et al. 2022).

According to Mori and Ito (2004), the caramel samples were diluted with water, adjusted the pH to 4.5 for analysis furanic compounds, and then treated it with SPE techniques. SPE technique with GC-MS yielded good recovery for the furanic compounds, from 76.2 to 101%. In the direct inject practice, Li et al. (2022) dissolved the caramel sample in 10% alcohol as the stock solution (1.0 g/m) before injecting it in GC-MS. Moreover, a Solid-Phase Microextraction (SPME) technique followed by GC-MS was recently used, whereXu et al. (2023) extracted the sample with DVB / Carboxen / PDMS fibre in the headspace vials. First, Xu et al. incubated the sample at 60 °C for 5 min, then adsorbed volatile samples using the fibre for 4 min. Followed by desorption the fibre in injector of GC for 1.5 min at 200 °C.

SPME is a pre-concentration technology that allows volatile chemicals to be extracted straight from the sample matrix. This is followed by separation and identification using GC-MS, a technique known for providing exact qualitative and quantitative data (Saison et al. 2009; Moreira et al. 2019). SPME combined with GC-MS exhibited exceptional durability, applicability across a broad spectrum of concentrations, commendable accuracy even for analytes measuring less than micrograms per litre. The advantage of SPME is it provides a free of solvents extraction method, reducing the possibility of sample contamination and eco-friendly method (Saison et al. 2009; Piergiovanni et al. 2023).

In the GC systems, the wax column and HP-5ms column were chosen for the analysis. Both columns are efficient for the separation and analysis of polar and non-polar furanic compounds. WAX column was high-polar polyethylene glycol-acid or polypropylene glycol-acid stationary phase is ideal for polar substance analysis (Mansour et al. 2015). Meanwhile, HP-5ms column contains a 5% phenyl and 95% methylpolysiloxane phase with low leakage and high thermal stability (up to 350 °C), is a non-polar column. With the low leakage properties, it is essential to detect trace levels of volatile compounds with high sensitivity. Even though HP-5ms is a non-polar column, but it still can be used to separate the non-polar and semi-polar compounds. The type of column is distinguished by its bonding, crosslinking, and inertness to active chemicals (Arukwe et al. 2012; McNair et al. 2019; Sani et al. 2020).

Optimization of numerous GC parameters such as inlet temperature, carrier gas flow rate, and temperature programme prior to analysis is important to improve the separation and detection. Based on Table 3, the inlet temperature was set at 200–230 °C. The carrying gas was Helium. The mode of the injector could be either split or spitless. The flow rate of the gas phase was 40–42 cm/s(Mori and Ito 2004) or 1 mL/min (Li et al. 2022). The total run time of the GC method was 34.35–53 min. It is a long analysis time, which may limit the throughput for large numbers of samples. Reported recovery spanned 76–101% (Mori and Ito 2004), indicating good accuracy and reliability.

In the GC-MS method, sample preparation steps like pH adjustment, and extraction can be time consuming and may cause errors or losses. For the SPME-GCMS method, it is limited to volatile and semi-volatile compounds, non-volatile compounds may not be effectively analysed by using GC method. The complex caramel matrix might interfere in the analysis, or it requires additional sample clean-up steps such as derivatization step (Castro et al. 2008; Gan et al. 2023).

#### Spectroscopic method

Spectroscopy is an effective method to identify HMF in food items by utilizing several electromagnetic radiation wavelengths, including visible, infrared, and ultraviolet (Pasias et al. 2017). It is also the most often used method for HPLC system detection. The chromophores in the HMF chemical structure make it possible to use the absorption approach. Moreover, selectivity has been increased by using chemical derivatization (Besir et al. 2021). In the research of Carabasa-Giribet and Ibarz-Ribas (2000), HMF was analysed in a UV-visible spectrophotometer at wavelength 280 nm after reacting with *p*-toluidine and barbituric acid. The goal of Carabasa and Ribas (2000) was not to create a technique for HMF detection or quantification. As a result, this article lacks information on the analytical features (precision, detection limits, and recoveries).

UV/Vis spectrophotometry is an ease of use and affordability (Cerdà et al. 2018). It complies with recommendations for green analytical chemistry. To increase selectivity and prevent matrix effects, sample pretreatment and/or analyte derivatization are necessary; yet these procedures consume time (Núñez and Lucci 2016). HPLC method gives more accurate data than the spectroscopic method, but the spectroscopic method is considered a cheap and easily applicable method (Besir et al. 2021).

### Factors affecting the formation of furanic compounds in caramels

Studies on non-enzymatic browning reactions in different model systems revealed the caramelization and the Maillard reaction’s complex dynamics. It highlighted that temperature, pH, type of sugar, amino acid, and additives affect furanic compounds formed in these processes.

Studies found lower temperature (Guan, Zhu, et al., 2011; Huang et al. 2016; Li et al. 2022; Lim et al. 2010), shorter heating time (Carabasa-Giribet and Ibarz-Ribas 2000; Chen et al. 2009), higher pH (Guan, Yu, et al., 2011; Huang et al. 2016; Mori and Ito 2004; Zhang et al. 2013) and lower sugar concentration (Chen et al. 2009; Dogan and Toker 2015; Liu et al. 2019) were effective to reduce HMF in caramel models.

The pH effect is critical; HMF levels rise dramatically in acidic situations at pH 1 to 5. During Maillard process, the Amadori compound is converted into 1,2-enaminol, which yields 3-deoxy-2-hexosulose while losing a water molecule to form HMF. Carbohydrate breakdown and isomerization at high temperatures can contribute to HMF production. A low pH promotes sugar breakdown by producing more reducing sugars and boosting HMF synthesis (Huang et al. 2016; Liu et al. 2019).

For the effect of irradiation on caramel model, Oh et al. (2005) found no HMF and 2-furfural in various type of caramel models and Zou et al. (2022) demonstrated the absence of HMF in glucosamine caramel models. Oh et al. used a cobalt-60 irradiator (γ-irradiation) to treat sugars (xylose, arabinose, fructose, glucose or sucrose) with or without lysine at 0, 5, 10, 20 and 30 kGy at room temperature, while Zou et al. used a UV-C LED source (275 nm) treated glucosamine solution for 0, 20, 40, 60, 80, 100 and 120 min. They found that higher irradiation dosages resulted in higher browning effects. Irradiation induces alterations in the physical and chemical properties of low molecular weight sugars and their derivatives, particularly in solid or aqueous states.

Regarding pressure conditions, Guan, Yu, et al. (2011) observed diminished HMF levels in glucose-ammonium sulphite caramel under higher-pressure cooking conditions. This phenomenon was ascribed to increase in dissociative sulphite content within caramel, inhibiting the degradation of Amadori rearrangement products. Dhungel et al. (2018) noted a reduction in HMF concentration in vacuum sous-vide glucosamine caramel compared to non-vacuum conditions, attributing this to the absence of oxygen during glucosamine caramelization.

Concerning reactants, citric acid, and oxalic acid, reduce HMF effectively in glucose-glutamic acid caramel (Lim et al. 2010). Both acids successfully reduce the HMF from 6.27 $\mu g/mL$ (control) to 5.4 $\mu g/mL$ after 4 weeks storage in the air at 30 °C. Lim et al. postulates the potential utilization of these acids in soybean paste for extended room-temperature storage. Ammonium sulphite emerges as a significant inhibitor of HMF in glucose caramel, as reported by Guan, Zhu et al. (2011), elucidating sulphite’s role in limiting the formation of N-glucosamine, a precursor to HMF. Zhang et al. (2013) studied the impact of polyphenols on the formation of HMF and furfural in fructose caramel model. They found that amount of furfural only tenth of HMF and only rosmarinic acid concurrently diminishing HMF quantities.        

Dietary fibres, like high-performance inulin and high-performance for high-temperature process inulin, significantly inhibit HMF in sucrose caramel. These inulins find potential application in the production of jams and fruit gels or other food products, contributing to the creation of products with enhanced prebiotic effects and reduced HMF content (Dogan and Toker 2015). Histidine emerges as an effective mitigator of HMF content in fructose caramel, attributed to its involvement in pigment formation during the final stages of the Maillard reaction in fructose-histidine caramel (Liu et al. 2019). Gallic acid demonstrated effectiveness in mitigating HMF in glucose-arginine (49%) and sucrose-arginine (54%) models, acting to prevent the oxidation of Maillard reaction intermediates (Abrantes et al. 2022).

Conversely, reactants like NaCl induce the heightened formation of HMF in glucose model. Kocadaǧll and Gökmen (2016) found that NaCl increases 4-fold HMF in glucose model. HMF is predominantly formed through fructose dehydration, and salt increases the rate constants for fructose production. Salt-induced hydrolysis of sucrose into glucose and fructose accelerates caramelization, enhancing HMF production (Kocadaǧll and Gökmen 2016). In the finding of Xu et al. (2023), it was also found that NaCl and KCl significantly increased the formation of furfural, 5-methylfurfural and HMF.

## Conclusion

This review summarizes and discusses the furanic compounds in caramel model. Furanic compounds in caramel models have drawn interest because of health and food quality issues.

In the caramel model, glucose and lysine were the most selected sugar and amino acids. HMF is the main furanic compound across the studies. It is an indicator and essential intermediate for the caramel model. For the caramel preparation, researchers preferred temperatures at 50–90 °C, and the duration was based on the temperature selected. The preferred pH was 6.5–7.5, a neutral caramel. HPLC is the predominant method to determine furanic compounds in the caramel models. It is because the sample does not need to be derivatized and is easy to prepare by using HPLC method as compared to GC method.

The number of HMF in the caramel model varies depending on the type of sugar, type of amino acids, temperature, duration of heating, additives added, pressure and pH of the model. In caramel models, lower temperature, shorter heating time, higher pH, and lower sugar concentration will reduce HMF production. Other parameters including irradiation, pressure, specific reactants, and additives affect HMF synthesis, providing insights for strategic food system control and setting the groundwork for creative food business techniques.

This study consolidates major trends, methodological discoveries, and knowledge gaps in this specialised topic to reveal foundational research and applied innovation on caramelization process optimisation for food quality, safety, and health. Standardised caramel model development, detection technique improvements, and harmful residue-reducing reactants are future research priorities in food industry.

## Data Availability

The datasets used and/or analysed during the current study are available from the corresponding author on reasonable request.

## Abbreviations

GC-MS: Gas chromatography/Mass Spectrometry
HPLC: High performance liquid chromatography
HMFO: 4-hydroxy-5-methyl-3(2 H)-furanone
HMF: 5-hydroxymethyl-2-furfural
KCl: Potassium chloride
LOD: Limit of detection
LOQ: Limit of quantitation
NaCl: Sodium chloride
PRISMA: Preferred Reporting Items for Systematic Reviews and Meta-Analyses
QuEChERS: Quick, Easy, Cheap, Effective, Rugged and Safe
SLR: Systematic literature review
SMF: 5-sulfoxymethylfurfural
SPME: Solid-phase microextraction
SULT: Sulfotransferases
UV: Ultraviolet
Vis: Visible
